# Co-Detection of VEGF-A and Its Regulator, microRNA-181a, May Indicate Central Nervous System Involvement in Pediatric Leukemia

**DOI:** 10.3389/pore.2022.1610096

**Published:** 2022-04-05

**Authors:** Bálint Egyed, Anna Horváth, Ágnes F. Semsei, Csaba Szalai, Judit Müller, Dániel J. Erdélyi, Gábor T. Kovács

**Affiliations:** ^1^ Hematology Unit, 2nd Department of Pediatrics, Semmelweis University, Budapest, Hungary; ^2^ Clinical Genomics Research Group, Department of Genetics, Cell- and Immunobiology, Semmelweis University, Budapest, Hungary; ^3^ HCEMM-SE Molecular Oncohematology Research Group, 1st Department of Pathology and Experimental Cancer Research, Semmelweis University, Budapest, Hungary

**Keywords:** cerebrospinal fluid, biomarkers, liquid biopsy, central nervous system involvement, pediatric leukemia, enzyme-linked immunosorbent assay

## Abstract

Central nervous system (CNS) involvement is a leading cause of therapy-refractory pediatric acute lymphoblastic leukemia (pALL), which is aggravated by underdiagnosing CNS disease with the currently used cell-based approach of cerebrospinal fluid (CSF) diagnostics. Our study focused on developing novel subcellular CNS leukemia indicators in the CSF and the bone marrow (BM) of patients with pALL. Serial liquid biopsy samples (*n* = 65) were analyzed by Elisas to measure the level of essential proteins associated with blast cell CNS trafficking, vascular endothelial growth factor A (VEGF-A) and integrin alpha 6 (ITGA6). In CSF samples from early induction chemotherapy, VEGF-A concentration were uniformly elevated in the CNS-positive group compared to those patients without unambiguous meningeal infiltration (9 vs Nine patients, Δc = 17.2 pg/ml, *p* = 0.016). Expression of miR-181a, a *VEGFA*-regulating microRNA which showed increased level in CNS leukemia in our previous experiments, was then paralleled with VEGF-A concentration. A slight correlation between the levels of miR-181a and VEGF-A indicators in CSF and BM samples was revealed (*n* = 46, Pearson’s *r* = 0.36, *p* = 0.015). After validating in international cohorts, the joint quantification of miR-181a and VEGF-A might provide a novel tool to precisely diagnose CNS involvement and adjust CNS-directed therapy in pALL.

## Introduction

Pediatric acute lymphoblastic leukemia (ALL) is still the leading cause of cancer-related long-term disability influencing the quality of life not only in childhood but also in adulthood [1]. In ALL, a major clinical concern is to overcome central nervous system (CNS) infiltration which highly predisposes the patient to relapse with dismal prognosis [2]. Even then, we lack sensitive methods for diagnosing and monitoring CNS ALL as contemporary routine cytomorphology/cytometry/cytospin analysis of the cerebrospinal fluid (CSF) produce >40% false-negative reports. Risk-adapted CNS-directed therapy (intrathecal and intravenous chemotherapy plus craniospinal irradiation) is a considerable determinant of leukemia-free survival, even in patients without a detectable initial CNS manifestation [3]. However, possible neurotoxic side effects of CNS-directed treatment, such as neurocognitive impairment or secondary brain tumor, limit therapy intensification opportunities. Finding the individualized balance of CNS-targeted therapy in order to reduce both toxic events and relapse rates is a clinical challenge. A gold standard method for CNS leukemia staging, which can identify and monitor occult, subclinical meningeal involvement as well, need to be urgently developed.

Previous studies suggest that subcellular biomarkers in the CSF may help to identify undiagnosed CNS leukemia cases. Earlier, more than 30 humoral factors and a set of molecular markers have been tested in the CSF, although, most of these candidate markers were proved not to be eligible for diagnostics in real clinical cohorts [4]. Recently, information gained by the thorough examination of molecular mechanisms and pathobiology of blast cell trafficking to the CNS compartment in mouse models open new perspectives, and refers to the central role of vascular endothelial growth factor A (VEGF-A) and integrin alpha 6 (ITGA6) proteins in neural invasion [5,6]. Independently, our group found markedly elevated level of microRNA-181a (miR-181a), a leukemia-related miR, in CSF samples of patients with CNS infiltration [7]. While the relationship between miR-181a and ITGA6 is only a possibility which is proposed by integrative miR target predictor databases, direct data suggest the regulative effect of miR-181a on VEGF-A level [8].

To establish a clinical diagnostics study on CNS leukemia with state-of-the-art candidate subcellular biomarkers, we analyzed serial liquid biopsies from the CNS and bone marrow (BM) niches in children with ALL by measuring VEGF-A and ITGA6 concentrations and correlating these data with miR-181a expressions.

## Methods

### Patient Cohort and Samples

The study is based on patients (aged ≤ 18 years) diagnosed with ALL in two Hungarian pediatric hematology centers (Semmelweis University second Department of Pediatrics and Heim Pal National Pediatric Institute). We collected CSF and BM of patients at diagnosis and at day 15 of the induction cycle of the ALL IC-BFM 2009 trial protocol, between October 2015 and August 2019. Consecutive lumbar punctures are the standard of care for evaluating leukemic CNS involvement depending on cytomorphology of the CSF. In the treating centers mentioned above, CSF samples were analyzed by cell counting chamber and cytospin preparate to assess the initial CNS status of the patient (examination results are available in Supplementary File S1). Within 2 h of sampling, preparation platelet-free plasma (PFP) from BM samples were carried out by centrifugation at 2500 *g* for 15 min two times, while cell- and debris-free CSF samples were gained after centrifugation at 300 g for 10 min. Processed BM PFP and CSF samples were then stored at −80°C. In our study, we examined CNS-positive (CNS^+^, i.e. unambiguously identified blasts by cytological techniques) ALL (*n* = 9) and matched CNS-negative (CNS^−^, i.e. blast-free by cytological methods) patients (*n* = 9) based on sex, age at the diagnosis and immunophenotype. In addition, patients (*n* = 6) diagnosed with spinal muscular atrophy (SMA) were selected as CSF reference samples of non-cancerous origin. Further information about the patient cohort is available in Table 1 and Supplementary File S1.

**TABLE 1:** Baseline population characteristics according to central nervous system status.

	CNS^+^ group	CNS^−^ group
Cases, *n*	9	9
Average age, *yr* (range)	6.9 (0.7–18.9)	10.7 (2.7–17.8)
Males, *%*	67	44
Disease progression state, *n*		
*De novo* ALL	7	8
Relapsed ALL	2	1
Immunophenotype, *n*		
Pre-B cell	4	1
Common (precursor B-cell)	3	6
Pre-T cell	1	1
Medullary T-cell	1	0
Mature T-cell	0	1
Sample type and sampling day, *n*		
CSF, day 0	8	9
CSF, day 15	8	8
BM PFP, day 0	7	9
BM PFP, day 15	7	9

CNS, central nervous system; ALL, acute lymphoblastic leukemia; CSF, cerebrospinal fluid; BM, bone marrow; PFP, platelet-free plasma.

### Protein Concentration Measurements

ELISA was used for quantitative detection of VEGF-A (VEGF-A Human ELISA Kit, Thermo Fisher Scientific, Waltham, MA, United States) and ITGA6 (Human ITGa6 ELISA Kit, Biorbyt, St. Louis, MO, United States) in accordance with the manufacturer’s guidelines. Due to the fact that we expected low protein concentrations, we transferred 75 µl of the samples to each well instead of the recommended 50 µl to assure a significant absorbance when performing the VEGF-A assay. The ITGA6 measurement was performed according to the manufacturer’s instructions, with a volume of 100 µl per sample. Both ELISA kits contained standard samples which were serially diluted to generate a calibration curve. ELISA reactions were processed on pre-coated plates and spectrophotometry was applied to read the final optical density values.

### Determination of microRNA-181a (miR-181a) Expression

In our previous study, we aimed to detect and quantify miR-181a levels in BM PFP and CSF samples of the same patient cohort [7]. After separating and pre-processing the miR fraction as detailed earlier [7], TaqMan Advanced miRNA Assays and TaqMan Fast Advanced Master Mix (Thermo Fisher Scientific, Waltham, MA, United States) were used to perform qPCR reactions. Measurements were carried out on the 7900HT Fast Real-Time PCR System (Thermo Fisher Scientific, Waltham, MA, United States). Comparative cycle threshold algorithm was applied to estimate normalized miR-181a expression, in which non-leukemic SMA samples were used as reference specimen.

### Statistical Analysis

All statistical analyses were performed using R version 4.0.3 (R Foundation for Statistical Computing, Vienna, Austria). In order to determine the concentration of the analyte within a sample, we used the non-linear 4-Parameter Logistic (4 PL) curve model. We fit the data obtained for the standard samples to the 4 PL curve by Levenberg-Marquardt algorithm (drc package). Concentration estimates were calculated as the natural logarithm of VEGF-A or ITGA6 absolute levels for each sample. Linear regression models were used to assess the statistical significance between each subgroup, where gender, age at diagnosis and ALL immunophenotype were considered as cofactors. The correlations between the expression level of miR-181a and the estimate of concentration was compared using Pearson’s method and Student’s t-test. Alpha level of 0.05 was used as criterion for statistical significance after false discovery rate correction, where it was relevant.

## Results

### Patient Characteristics

ELISA measurements were performed on 33 CSF and 32 BM PFP samples collected from 18 children with ALL and on CSF reference samples from six SMA patients. The baseline characteristics for this cohort is described in Table 1. Briefly, the median age was 6.4, more than three-quarter of patients had precursor B-cell immunophenotype, and various genetic subgroups were involved (normal karyotype, hyperdiploid, high hyperdiploid, *KMT2A*-rearranged, *P2RY8*-*CRLF2* translocated, *CDKN2A* deleted and *ETV6* deleted).

### Soluble VEGF-A and ITGA6 Levels in CSF and BM Samples of Children With ALL

To evaluate the VEGF-A and ITGA6 content of cell-free CSF and platelet-free BM plasma samples from both CNS^+^ and CNS^−^ patients, two commercial ELISA tests were used. Due to the paucity of cell-derived material in CSF compared to other body fluids, we questioned whether these proteins can be detected in CSF supernatant. VEGF-A was measurable in all CSF samples in the CNS^+^ as well as the CNS^−^ group, as seen in Table 2. However, the concentrations were markedly lower in the CSF relative to the BM PFP, independent of CNS status (for detailed description, *see* Table 2). This CSF–BM gap in VEGF-A concentration was almost 2-fold greater in the CNS^−^ patients compared to the CNS^+^ group. As opposed to these results, ITGA6 was not detectable in our CSF or BM samples using this method.

**TABLE 2:** Vascular endothelial growth factor A (VEGF-A) and microRNA-181a (miR-181a) concentrations measured in different sample types of patients with and without CNS leukemia.

	CNS positive group			CNS negative group		
*VEGF-A*	c (pg/ml)	Avg. ∆c (pg/ml)^a^	*p* value^a^	c (pg/ml)	Avg. ∆c (pg/ml)^a^	*p* value^a^
BM PFP d0	131.2 ± 43.4	—	—	130.6 ± 22.7	—	—
BM PFP d15	179.7 ± 93.0	—	—	145.6 ± 30.1	—	—
CSF d0	44.4 ± 4.0	47.9	0.030	24.8 ± 1.8	90.14	<0.001
CSF d15	45.2 ± 3.3	61.4	0.011	31.7 ± 3.7	86.7	0.001
***miR-181a***	**FC**	**Avg. ∆FC^a^**	***p* value^a^**	**FC**	**Avg. ∆FC^a^**	***p* value^a^**
BM PFP d0	34.8 ± 13.1	—	—	32.9 ± 13.2	—	—
BM PFP d15	1.5 ± 1.1	—	—	0.5 ± 0.1	—	—
CSF d0	14.9 ± 5.5	25.99	0.141	1.6 ± 0.8	33.1	0.018
CSF d15	3.1 ± 1.3	TLN	TLN	0.2 ± 0.2	TLN	TLN

Concentration and fold change data are displayed as mean ± standard error of the mean. CNS, central nervous system; BM, bone marrow; CSF, cerebrospinal fluid; PFP, platelet-free plasma; FC, fold change; Avg., average; TLN, too low number of patients to statistically analyze.

a In-patient VEGF-A concentration and miR-181a expression fold change differences between CSF and BM PFP samples (c_BM_–c_CSF_ and FC_BM_–FC_CSF_, respectively) were averaged. *p* values were calculated by linear regression to compare CSF and BM PFP VEGF-A levels and miR-181a expressions.

**FIGURE 1:**
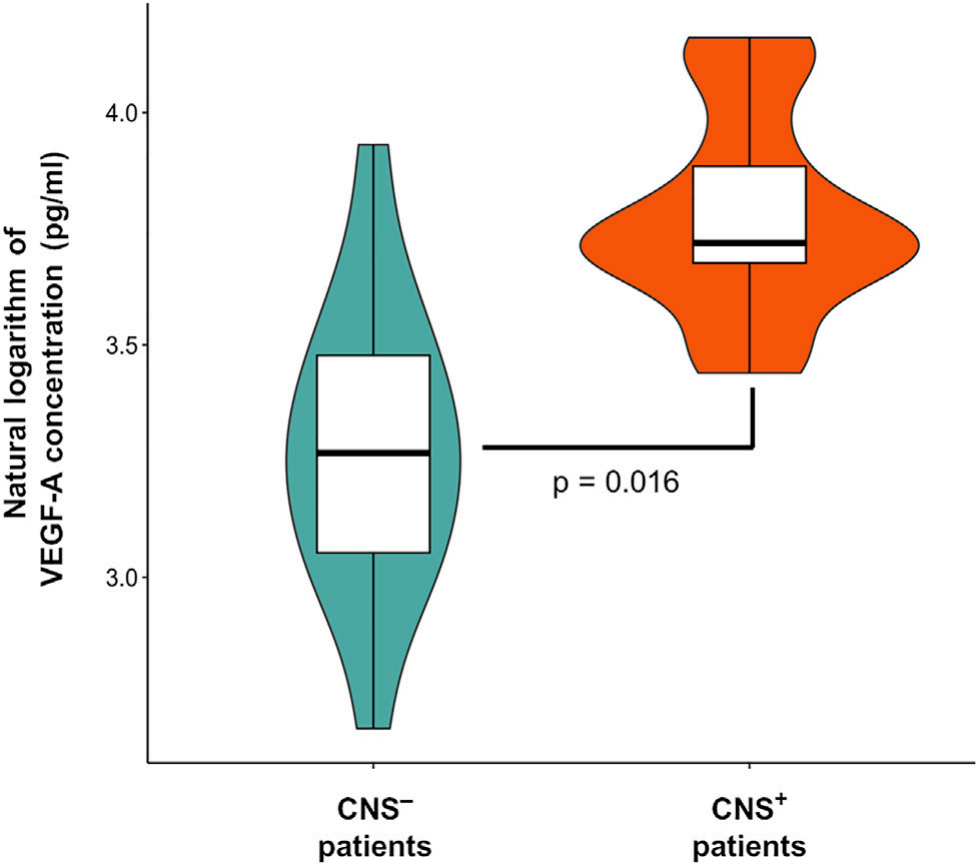
Vascular endothelial growth factor A (VEGF-A) levels in the cerebrospinal fluid according to central nervous system (CNS) status in pediatric acute lymphoblastic leukemia. Violin plot describes the distribution of VEGF-A data. Box plot’s box indicates mean ± SD, whiskers are means ± 3 SD.

VEGF-A concentration estimates (natural logarithm values) were uniformly elevated in the CNS^+^ ALL group compared to CNS^−^ patients when analyzing CSF samples from day 0 and day 15 of the induction chemotherapy (Δc = 17.15 pg/ml, *p* = 0.016). This means a mildly more than 1.5-fold difference in concentrations between the two subgroups (mean value in CNS^+^ vs CNS^−^ patients: 43.61 vs 26.45 pg/ml), as seen in Figure 1. Gender, age at diagnosis and ALL immunophenotype were not significant contributors in the regression model. In this limited CNS^+^ cohort, we were not able to demonstrate a change in VEGF-A level between d0 (rich in blasts) and d15 (paucity of blast cells) CSF samples. However, change in BM VEGF-A levels from d0 to d15 was not proportional to flow cytometry-based BM residual disease dynamics (Supplementary File S1), so the linear correlation between VEGF-A concentration and ALL cell burden remains unproven.

### Correlation of VEGF-A Level and miR-181a Expression in Liquid Biopsy Samples From Leukemia Niches

**FIGURE 2:**
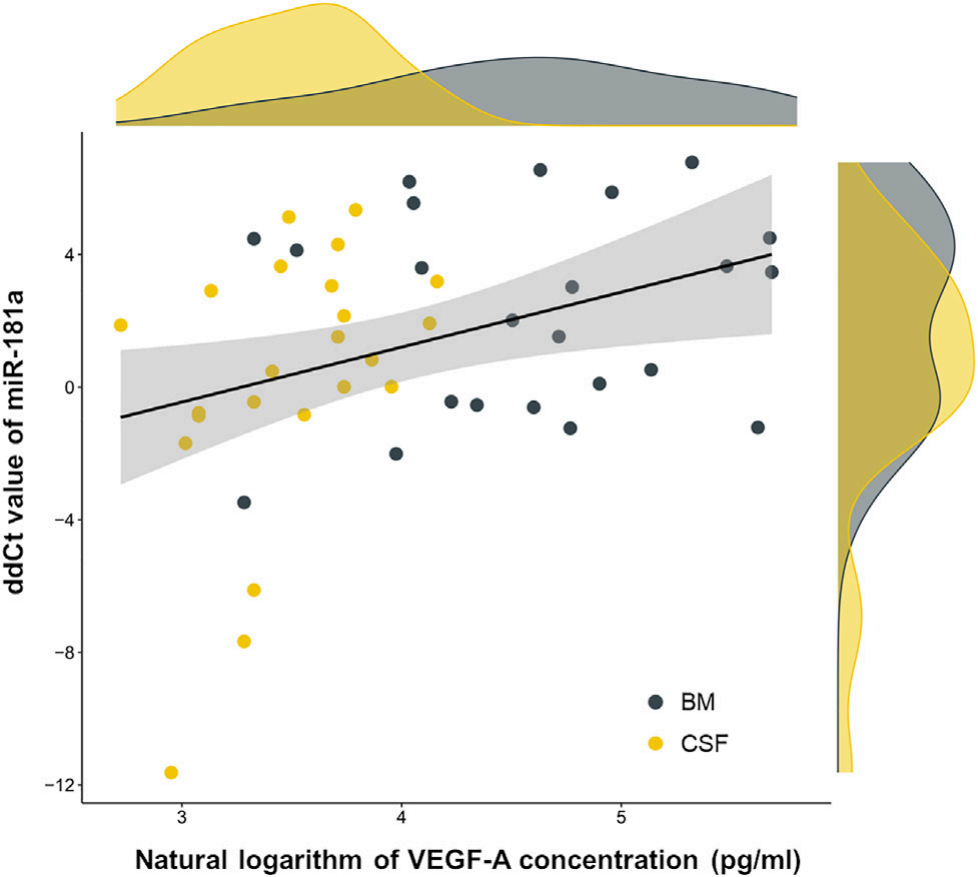
Co-detection of two possible subcellular central nervous system leukemia indicators, microRNA-181a and vascular endothelial growth factor A (VEGF-A). Linear correlation (black line) was fitted on dots representing patient samples. Marginal density plots describe sample distributions. Yellow color represents cerebrospinal fluid (CSF) samples, grey color marks bone marrow (BM) platelet-free plasma samples. Abbreviation: miR, microRNA.

After the identification of VEGF-A concentration gap between CSF samples of CNS^+^ and CNS^−^ ALL patients, we addressed whether VEGF-A levels correlates with a previously proposed marker of CNS leukemia, miR-181a. Relative miR-181a expressions in the same patient cohort were determined by delta-delta cycle threshold (ddCt) algorithm as we previously published [7]. As of interest, miR-181a and VEGF-A indicators–i.e. ddCt value and natural logarithm of the concentration, respectively–in CSF and BM PFP samples from day 0 and day 15 of the treatment showed a slight correlation (*n* = 46, Pearson’s *r* = 0.36, *p* = 0.015), as displayed in Figure 2.

## Discussion

As the identification and follow-up of pediatric ALL manifestation in the CNS compartment present difficulties in current clinical practice, sensitive and easy-to-measure novel markers are needed to develop. Our study proposes a subcellular approach of CNS leukemia diagnostics. We measured VEGF-A and ITGA6 concentrations in CSF samples of children with CNS^+^ and CNS^−^ ALL defined based on cytomorphologic examinations. The CSF level of VEGF-A was significantly elevated in patients with meningeal disease compared to those without CNS involvement. The role of VEGF-A was previously investigated in preclinical models of CNS leukemia. Münch et al showed that patient derived ALL cells transplanted onto mice invaded the CNS niche by transmigrating through microvascular endothelial cells facilitated by VEGF-A signaling [5]. Similar mouse models were used in a report by Kato et al depicting the hypoxia-responsive gene *VEGFA* upregulation in the nutrient-poor CNS microenvironment [9]. However, clinical data are insufficient to draw conclusions regarding the CNS leukemia indicator role of VEGF-A. RNA sequencing of primary leukemic cells isolated from the cerebrospinal fluid of a small children cohort with CNS involvement revealed markedly elevated *VEGFA* expression in these cells [9], but CNS^−^ samples were not analyzed parallelly. In a study with pediatric and adult acute leukemia patients, levels of soluble VEGF-A receptors and their ratio to VEGF-A concentration in CSF and serum were proposed as a prognostic marker of leukemia and a contributor of CNS involvement evolution [10]. Nevertheless, our study provides the first description of the significance of VEGF-A ELISA measurements in leukemic CSF samples in CNS leukemia identification.

Our VEGF-A concentration results in BM PFP samples showed no difference between day 0 and day 15 of the treatment (Table 1), however, BM measurable residual disease uniformly decreased to the 15th day of chemotherapy (Supplementary File S1). This suggest that VEGF-A level is not an indicator of BM leukemic burden, while we propose CSF VEGF-A concentration as a marker of ALL expansion in the CNS compartment. This seemingly contradictory situation can be resolved by the above cited evidences claiming that VEGF-A production in ALL cells is predominantly evoked by the hypoxic environment, e.g. in the CSF niche. If we consider that higher initial CSF VEGF-A level may indicate a biological subtype of ALL with high risk for permanent meningeal blast deposits (and not circulating leukemic burden in the CSF), later relapses could be expected and fueled by these dormant CNS clones. A long-term follow up of our patients for late BM and/or CNS relapses would be beneficial to analyze this question. Case reports confirming the clonal role of hidden, long-surviving CNS blasts behind later relapses in any of the main leukemic body compartments are available [11].

The positive correlation between VEGF-A level and regulatory RNA miR-181a expression in BM PFP and CSF samples of childhood ALL cases was also observed. MiR-181a seemed to be a sensitive subcellular marker of CNS disease in our previous study [7]. There is potential evidence explaining the relationship of miR-181a and VEGF-A in cancer. In human chondrosarcoma cell lines, miR-181a transfection raised the expression of VEGF messenger RNA and increased secreted VEGF protein quantity [12]. An in-depth study on how VEGF signaling is promoted by miR-181a in preclinical models of colorectal cancer identified SRC kinase signaling inhibitor 1 (SRCIN1), an angiogenesis suppressor, as a key target of miR-181a [8]. Studies investigating whether these mechanisms work in ALL as well are lacking. Interestingly, we did not see intra-patient reduction in BM or CSF VEGF-A concentrations to the 15th day of chemotherapy, while miR-181a levels significantly decreased in both body compartments in both CNS^+^ and CNS^−^ groups [7]. If we suspect a direct miR-181a‒VEGF-A regulatory relationship, a fall in VEGF-A levels would be expected as well. However, generally, miR expression level does not necessarily correlate with the target mRNA‒protein levels since mRNA expression and protein translation may be regulated in several different molecular ways. Our data (Figure 2) show the same situation regarding the correlation of miR-181a expression and VEGF-A concentration as there was no straightforward reciprocity in the regulatory miR and protein levels. A hypothesis can be that miR-181a, an early marker of initial CNS leukemia burden, is produced when blasts recognize the nutrient-poor, hypoxic CSF microenvironment, and the consecutive increased excretion of VEGF-A is a prolonged phenomenon indicating persistent subclinical meningeal manifestation, but this approach definitely needs further and deeper investigation.

ITGA6 was not measurable in cell-free ALL samples by the ELISA method we used. ITGA6-expressing blasts are actively engaged in adhesion and migration on the abluminal side of vessels to the CNS as multiple studies suggest [6,13]. The absence of ITGA6 in BM and CSF supernatants gained by cell removal in our study claims that this cell surface protein of blast cells may not be likely to be involved in integrin shedding or such process allocating ITGA6 into the extracellular space. Previously, high ITGA6 expressions were measured inside the CNS-invading leukemic cells [14].

In summary, our data represents the first clinical observation of VEGF-A based CNS status evaluation and the identification of VEGF-A and miR-181a co-expression in ALL. However, several limitations should be considered when interpreting our results. The rarity of CNS involvement in childhood leukemia resulted in low patient numbers. Another bias is the selection of patients without CNS disease, which relied on insensitive conventional diagnostics and carries the risk of involving patients with occult CNS involvement in the CNS^−^ group. Yet, this study provides the rationale for future studies of subcellular elements, soluble proteins and miRs, in the diagnostics of CNS leukemia.

## Data Availability

The original contributions presented in the study are included in the article, further inquiries can be directed to the corresponding author.
